# Effect of bio-engineering on size, shape, composition and rigidity of bacterial microcompartments

**DOI:** 10.1038/srep36899

**Published:** 2016-11-15

**Authors:** Matthias J. Mayer, Rokas Juodeikis, Ian R. Brown, Stefanie Frank, David J. Palmer, Evelyne Deery, David M. Beal, Wei-Feng Xue, Martin J. Warren

**Affiliations:** 1 Industrial Biotechnology Centre and School of Biosciences, University of Kent, Giles Lane, Canterbury, Kent CT2 7NJ, UK; 2 Department of Biochemical Engineering, University College London, Bernard Katz Building, Gordon Street, London WC1E 6BT, UK

## Abstract

Bacterial microcompartments (BMCs) are proteinaceous organelles that are found in a broad range of bacteria and are composed of an outer shell that encases an enzyme cargo representing a specific metabolic process. The outer shell is made from a number of different proteins that form hexameric and pentameric tiles, which interact to allow the formation of a polyhedral edifice. We have previously shown that the *Citrobacter freundii* BMC associated with 1,2-propanediol utilization can be transferred into *Escherichia coli* to generate a recombinant BMC and that empty BMCs can be formed from just the shell proteins alone. Herein, a detailed structural and proteomic characterization of the wild type BMC is compared to the recombinant BMC and a number of empty BMC variants by 2D-gel electrophoresis, mass spectrometry, transmission electron microscopy (TEM) and atomic force microscopy (AFM). Specifically, it is shown that the wild type BMC and the recombinant BMC are similar in terms of composition, size, shape and mechanical properties, whereas the empty BMC variants are shown to be smaller, hollow and less malleable.

Some bacteria have an inherent ability to compartmentalize specific metabolic processes by encasing them within a proteinaceous organelle called a bacterial microcompartment (BMC)^1^,^2^,^3^. In so doing they generate cytoplasmic supramolecular complexes that look similar to phage, with a diameter of between 100–200 nm, when thin sections of BMC-containing bacteria are viewed by transmission electron microscopy^4^,^5^,^6^. BMCs consist of an outer semi-permeable protein shell that envelopes and secedes enzymes and metabolites from the main cellular milieu^7^,^8^,^9^,^10^,^11^. They are associated with either anabolic processes such as carbon fixation (carboxysomes)^5^ or with catabolic processes connected with the utilization of either carbon or nitrogen (metabolosomes)^12^. Carboxysomes house ribulose-1,5-bisphosphate carboxylase/oxygenase (RuBisCO) and carbonic anhydrase and act to promote a higher local concentration of carbon dioxide in order to increase the efficiency of the carboxylation reaction^13^. Metabolosomes are associated with fermentative processes with substrates such as ethanolamine, choline, fucose, rhamnose or 1,2-propanediol^1^. A recent bioinformatics analysis has indicated that there are at least 23 distinct BMCs found across a range of bacteria phyla^14^.

Of the metabolosomes, the best characterized is the 1,2-propanediol (1,2-PD) utilizing organelle (Fig. 1) from organisms such as *Salmonella enterica*^4^,^15^,^16^ and *Citrobacter freundii*^17^. The genetic information for the organelle is found within the propanediol utilization (*pdu)* operon, which contains 23 genes that encode for not only the shell proteins of the BMC but also the enzymes associated with the transformation of 1,2-PD into propionaldehyde and its subsequent disproportionation into propionic acid and propanol^4^. This includes the adenosylcobalamin-dependent diol dehydratase^4^,^16^ as well as enzymes linked to the reactivation of the coenzyme^18^,^19^,^20^. In this respect the metabolosomes house a more complex repertoire of enzymes than the carboxysomes and have a broader requirement for coenzymes and cofactors. The protein shell of the metabolosomes is proposed to protect the cytoplasm from toxic aldehyde intermediates^21^.

Structural data from X-ray studies of individual shell proteins from both carboxysomes and metabolosomes have revealed that they form hexameric-shaped tiles with a central pore that piece together to form the faces of the organelle whereas pentameric tiles act as the vertices of the structure^9^,^10^,^22^,^23^,^24^,^25^,^26^,^27^,^28^,^29^,^30^. The Pdu BMC contains 7 shell proteins, PduA, PduB, PduB’ PduJ, PduK, PduT and PduU, which generate the hexagonal tiles that represent the facets of the polyhedral structure (Fig. 1). Most of the shell proteins contain a single BMC domain that consists of about 90 amino acids, which fold into a β-α-β motif allowing them to aggregate as hexamers^9^. However, PduB, its N-terminally truncated derivative from an alternative translation start site (PduB’) and PduT contain tandem BMC domains that generate a pseudohexamer formed by a trimeric structure^23^,^27^,^28^. The vertices of the BMC are thought to be formed from the pentameric PduN^29^,^30^,^31^.

All shell proteins contain central pore(s) (Fig. 1), some of which appear to have alternative conformations^22^,^23^,^26^,^27^. Passage of substrates, products and cofactors across the shell is thought to be mediated by diffusion via the pores within the hexameric components of the shell face, movement that may be facilitated by a differential charge distribution of amino acid residues surrounding the pore. It is envisaged that engineering of these pores may change the ability of the BMC to allow passage of substrates, products or cofactors into or out of the macromolecular structure^32^,^33^ as has recently been evidenced through the manipulation of PduA pores^34^. Overall, the structure of the BMC has to be flexible enough to allow shell assembly and closure but also stable enough to maintain structural integrity. However, to date there is little information on the structural rigidity of these complexes.

BMCs represent genetically tractable systems that can be successfully cloned into organisms in which they are not normally found. In this respect, the Pdu system from *C. freundii* has been engineered into *E. coli* to allow for the formation of functional recombinant organelles^17^,^35^,^36^. Similarly, reports demonstrate that the ethanolamine utilization (Eut) system can be manipulated to allow for recombinant BMC production and protein targeting^37^,^38^. Recombinant carboxysomes, too, have been produced in *E. coli*, generating structures that are capable of carbon dioxide fixation^39^. Empty *C. freundii* Pdu microcompartments (eBMCs) have been formed from the coordinated recombinant production of PduA, B, B’, J, K, N and U^36^. However, the composition, structure and physical properties of purified wild-type BMCs (wtBMCs) have not been characterized in detail and compared to the full recombinant Pdu BMC (rBMC) or its empty derivatives. Herein, we compare the shell protein constituents of wtBMCs, rBMCs and eBMCs, and investigate their composition, form and nano-mechanical properties.

## Results

### *In vivo* characterization of recombinant and wild type Pdu BMCs

In order to compare wild type BMCs with recombinant and empty BMCs, *in vivo,* the sizes of the various bacterial organelles were first compared *in situ* after thin sectioning and visualization of the host bacteria by transmission electron microscopy (TEM) (Supplementary Figure 1). In this respect wild type Pdu BMCs (wtBMCs) from *C. freundii* were compared initially to recombinant *C. freundii* Pdu BMCs (rBMCs) produced in *E. coli.* The rBMC construct includes all shell proteins, metabolic enzymes for 1,2-PD degradation and reactivation factors for adenosylcobalamin. Subsequently, the wtBMC and rBMC were compared to a range of engineered empty recombinant BMCs (eBMCs) that were produced as a result of (i) the expression of the genes encoding all the shell proteins PduA-B-J-K-N-U-T (A-T), (ii) a reduced set of shell proteins PduA-B-J-K-N-U (A-U), and (iii) an empty BMC containing a fluorescent protein fused to PduA, mCherry-PduA-B-J-K-N-U (mA-U).

Growth of *C. freundii* in the presence of 1,2-propanediol resulted in the production of Pdu wtBMCs, which were easily visualized after embedding and thin sectioning of the bacteria. As expected, the wtBMCs appeared as heterogeneous polyhedral bodies that were evenly dispersed around the cell, as had been previously observed with the Pdu BMCs that are found in *S. enterica*^4^ and *L. reuterii*^40^. In contrast, rBMCs were observed densely packed in thin-sectioned *E. coli cells* harboring a plasmid containing the *pdu* operon. In this case the rBMCs take up the majority of available space within the cytoplasm of the cell. However, despite the difference in the quantity of organelles within the cell, both the wtBMCs and rBMCs were found to be of a similar size distribution.

When the three eBMCs variants (A-T, A-U and mA-U) were compared to one another, no phenotypical differences were observed in the TEM images of the thin-sectioned *E. coli* strains containing the eBMC constructs. All contained heterogeneous polyhedral bodies that were crammed into the cytoplasm of the cell. However, overall, all three eBMCs variants appeared smaller in diameter in comparison to the complete BMCs (wtBMCs and rBMCs, for images see Supplementary Figure 1).

### Purification of intact wtBMCs, rBMCs and eBMCs

In order to characterize and compare the structural and biochemical properties of wtBMCs, rBMCs and eBMCs, a number of purification methods to isolate intact BMCs were investigated involving the use of mild detergent lysis procedures, employing either a bacterial specific lysis reagent (B-PER™) or a yeast specific lysis reagent (Y-PER™). The bacterial protein extraction (B-PER™) method is based on lysing cells with B-PER™ and entails consecutive centrifugation steps to separate cell debris from Pdu metabolosomes^41^. This procedure was found to work best for the isolation of complete BMCs (wtBMCs and rBMCs). The yeast protein extraction reagent (Y-PER™) method is based on lysis of bacterial cells and subsequent salt precipitation and was found to be effective for the purification of eBMCs^35^. The purification and isolation of the various BMCs was followed by SDS-PAGE (Supplementary Figure 2). As the eBMCs variants were found to purify under different conditions to the rBMC and wtBMCs it was concluded that they must have different physical and chemical properties.

### Determination of the shell protein composition of wtBMCs, rBMCs and eBMCs

The purification of wtBMCs, rBMCs and eBMCs allowed their shell protein composition to be compared through a proteomic approach. Such holistic analysis gives information on the composition of the building blocks of these supramolecular structures, which is essential in order to understand their assembly and mechanical properties, as well as their ability to acquire substrates and cofactors. Differences in the shell protein composition of purified wtBMCs, rBMCs and eBMCs were examined by 2D-PAGE (Supplementary Figure 3) and MALDI MS-MS tandem mass spectrometry in order to identify the individual building blocks and to quantify their relative abundance.

**Figure 2:**
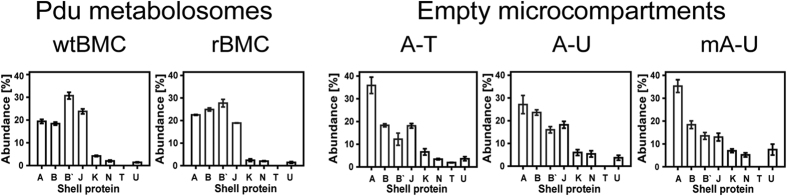
BMC shell protein composition. The shell protein composition is shown as a percentage of the total protein of wtBMC, rBMC and the eBMCs A-T, A-U, mA-U. The data represents the mean values of three independent repeats. The error-bars indicate one standard error of mean.

When the purified wtBMCs and rBMCs were analyzed by 2D-PAGE all the shell proteins could be detected by MALDI TOF-TOF. The abundance of the various shell proteins, in terms of per cent of total protein, was estimated by densitometry (Fig. 2). In wtBMCs, PduB’ (31%) was the most abundant shell protein followed by PduJ (24%), PduA (19%) and PduB (18%). In contrast, PduK, PduN, PduT and PduU were found to be minor components of the complex. PduT was not quantified due to its low spot intensity. The minor shell components were estimated to account for less than 8% of the capsid in total. In rBMCs the order of shell protein abundance is slightly different, with the order of PduB’ (28%) -PduB (24%) -PduA (22%) -PduJ (18%). The remaining shell proteins representing minor components of the structure. Thus, overall, the profile of the rBMC to the wtBMC is remarkably similar indicating that they have a very analogous composition, as expected. This analysis is also similar to that reported to the purified *S. enterica* Pdu BMC^16^.

As with the complete BMCs, all the encoded shell proteins of the purified eBMC variants could also be detected by MALDI TOF-TOF analysis of spots excised from 2D-PAGE. Surprisingly, in all three eBMC variants (A-T, A-U, mA-U) that were analyzed, as a percentage of the total protein, PduA was found to be the major Pdu shell protein component (27–35%), followed by PduB (18–24%), PduB’ (12–16%) and PduJ (13–18%) (Fig. 2). PduK, PduN, PduT (only in A-T) and PduU were all found to be minor shell components comprising less than 20% of the shell. Although some differences in the shell protein composition were noted for the eBMCs variants, the overall trend is similar (Fig. 2).

The major conclusion from this comparative proteomic analysis is that eBMCs appear to incorporate considerably more PduA relative to all other protein components, in comparison to their wtBMC and rBMC counterparts. Whether this reflects a different expression pattern or results from a lack of internal cargo has yet to be determined. However, it is likely that differences in shell protein composition will affect the nano-mechanical and functional properties of these bioengineered structures.

### Structural characterization by single particle analysis

**Figure 3:**
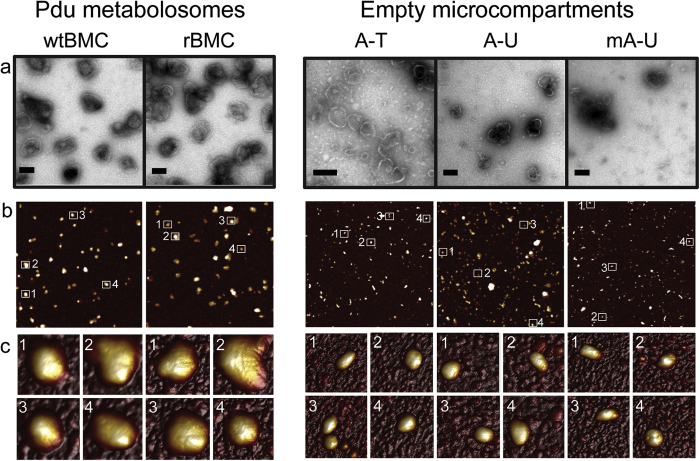
Comaprison and TEM and AFM images of isolated BMCs. (**a**) TEM images of purified wtBMCs, rBMCs and eBMCs (A-U, mA-U and A-T). The scale bars represent 100 nm. (**b**) Overview of zoomed-in 4 × 4 μm height AFM scans. White squares represent microcompartment particles shown in Fig. 3c. (**c**) 3D topology images of single zoomed in particles (240 × 240 nm each) from (**b**).

The structure/function relationship of BMCs is currently poorly understood but yet this knowledge is vital in order to understand and develop principles for engineering encapsulated processes^33^. To learn more about how variation in BMC composition alters the physical properties of the structures a combination of AFM and TEM (Fig. 3) were employed to image and characterize purified intact wtBMCs, rBMCs and eBMCs (A-T, A-U and mA-U).

When viewed by negative stain TEM, the isolated Pdu microcompartment samples (wtBMC, rBMC, A-T, A-U and mA-U) all appeared to be pure (i.e. not contaminated with other bodies) (Fig. 3). The TEM images therefore confirm the efficiency of the purification method used and are consistent with the proteomic analysis described above, which did not detect any major contaminating proteins. The isolated wtBMCs were observed to adopt a wide variety of different polyhedral shapes and sizes (Fig. 3) and appeared to be irregular in overall topology. No major differences in size and shape were visualized between the wtBMC and rBMC structures. This suggests that the heterologous formation of Pdu BMCs in *E. coli* does not affect BMC size. No detectable structural differences were visualized between the different eBMC variants. This suggests that the presence of the minor BMC shell building block PduT does not affect the BMC structure and that the fusion of mCherry to the N-terminus of the major BMC shell protein building block PduA does not disrupt BMC assembly, size or shape. However, as observed with the *in vivo* thin-section data, a clear size difference was observed between complete BMCs (wtBMCs and rBMCs) and the eBMC variants.

To complement the TEM data of the purified BMCs, AFM was also employed to provide both information on the 3D shapes of the structures and their nano-mechanical properties. The various microcompartments (wtBMC, rBMC, A-T, A-U, mA-U) were scanned in air using AFM. The general observations made by AFM are consistent with those made by TEM in that all of the isolated microcompartments appeared to be pure (i.e. not contaminated with other bodies). Both wtBMCs and rBMCs appeared heterogeneous in shape and size, but no size distribution differences were observed between the wtBMC and rBMC structures. The eBMC variants (A-T, A-U, mA-U) also appeared irregular in shape and heterogeneous in size. Despite the variation in size and shape of the eBMCs, the AFM analysis shows that they are indistinguishable in terms of their physical dimensions. As with the TEM imaging, both wtBMCs and rBMCs appear significantly larger than the eBMC variants when viewed by AFM (Fig. 3).

**Figure 4:**
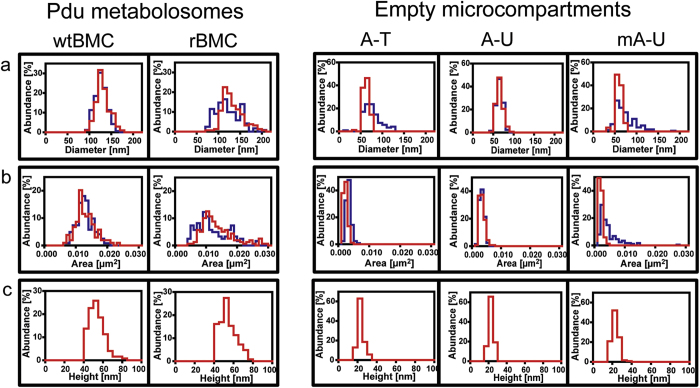
Comparative representation of diameter, area and height distributions of wtBMC, rBMC and eBMCs (A-T, A-U, mA-U). (**a**) Diameter distribution of normalized data of TEM (blue) and AFM (red), bin size is 10 nm. (**b**) Area distribution of normalized TEM (blue) and AFM (red) data, bin size is 0.001 μm^2^. (**c**) Height distribution of normalized AFM data, bin size is 5 nm.

From the TEM and AFM data sets that were collected, the diameter, apparent cross-sectional area and peak height of the wtBMCs and rBMCs were quantified and compared (Table 1). The diameter of the *C. freundii* wtBMCs was 127 ± 13.2 nm and 131 ± 15.4 nm as measure by TEM and AFM, respectively (Fig. 4, Table 1, Supplementary Table 1). The isolated rBMC structures showed a slightly wider variation (TEM: 122 ± 26.4 nm, AFM: 133 ± 23.6 nm). However, the average diameter size of the wtBMCs and rBMCs were alike (122–133 nm). Indeed, these average values of the *C. freundii* wtBMCs and rBMC are consistent with the reported average diameter of the Pdu BMCs that had been purified from *S. enterica* (123 ± 30 nm^10^).

To take the heterogeneous and irregular shape of Pdu BMCs into account, the apparent cross-sectional area occupied by wtBMC and rBMC particles were also quantified and compared (Table 1, Supplementary Table 2). The area occupied by individual wtBMCs was 0.0128 ± 0.0026 μm^2^ and 0.0137 ± 0.0032 μm^2^ as measured by TEM and AFM, respectively, whereas the single particle area of rBMCs was observed to be 0.0122 ± 0.0053 μm^2^ by TEM and 0.0143 ± 0.0053 μm^2^ by AFM. Therefore, as with the average diameter, rBMC particles displayed a higher variation in area size, although the average area size of wild-type and rBMCs showed no difference.

In addition to diameter and area quantification, the maximum height of particles can be measured by AFM. However, from the AFM scans of wtBMC and rBMC particles the maximum height of individual microcompartments was found to be smaller than the radius. This suggests that particle deformation may have occurred during sample deposition. Nonetheless, the maximum height information still provided new information concerning the size distribution of Pdu microcompartments. The peak height of wtBMCs and rBMC particles were both 50 ± 8 nm. As with both diameter and area, no significant differences were observed between the wtBMC and rBMC structures (Fig. 4, Table 1, Supplementary Table 3).

The application of AFM to the eBMC variants again highlighted a wide variety of sizes in terms of diameter (Table 1, Supplementary Table 1). Importantly, no substantial differences were observed for the average diameters of A-T, A-U and mA-U, ranging between 56 ± 8 nm and 77 ± 19 nm. The cross-sectional areas of all three tested eBMCs were also compared (Fig. 4, Table 1, Supplementary Table 2). Interestingly, no significant differences in area size between A-T, A-U and mA-U were identified. The vast majority of all tested eBMC variants had average values between 0.0025 ± 0.0007 μm^2^ and 0.0049 ± 0.0024 μm^2^. Finally, and as seen with the wtBMC and rBMC particles, the peak heights associated with the eBMC variants appeared distorted, as their peak heights were smaller than their diameter (Fig. 4, Table 1, Supplementary Table 3). The average peak height of the eBMC particles ranged from 18.4 ± 2.4 nm to 18.7 ± 3.3 nm. The average values of all three eBMC were similar with peak heights of 18–19 nm.

### Nano-mechanical mapping

**Figure 5:**
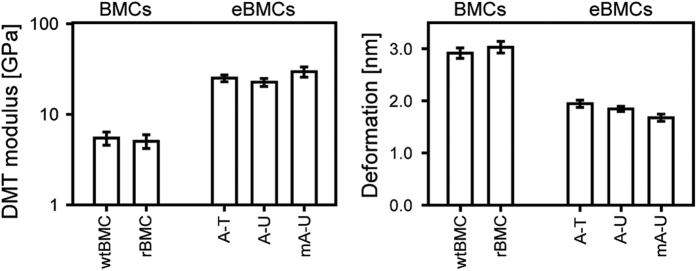
Quantitative nano-mechanical mapping of BMCs. The QMN mapping at 250 nN force set point for wtBMCs, rBMCs and eBMCs (A-U, mA-U, A-T). Average values and error-bars representing standard error of mean (of 20 measured particles) of reduced DMT modulus [GPa] and deformation [nm] are shown.

To further investigate the difference between complete BMCs (wtBMCs and rBMCs) and the eBMC variants (A-T, A-U and mA-U), the nano-mechanical properties of the microcompartments were quantified using AFM quantitative nano-mechanical mapping (QNM) (Bruker). Application of a force titration between 200 pN and 2 nN showed no deformation for any of the purified microcompartments and no change in the height of the compartments was observed, confirming that imaging of the microcompartments did not cause an observable distortion. However, in contrast to the low force experiments, QNM experiments at higher than 2 nN applied force showed significant nano-mechanical differences between complete BMCs and eBMCs (Fig. 5). At a force set point of 250 nN, both wtBMCs and rBMC showed an average maximum deformation of 2.9–3.0 nm, whereas eBMCs showed an average maximum deformation of 1.7–1.9 nm. The same QNM experiments also revealed that the reduced elastic modulus (E*) is on average 5.0–5.4 GPa for both wtBMCs and rBMCs, whilst eBMCs displayed values between 22.8–29.8 GPa. In summary, single particle AFM measurements show striking differences in size distribution and mechanical properties for wtBMCs and rBMCs in comparison to eBMCs.

### Internal structural organization

**Figure 6:**
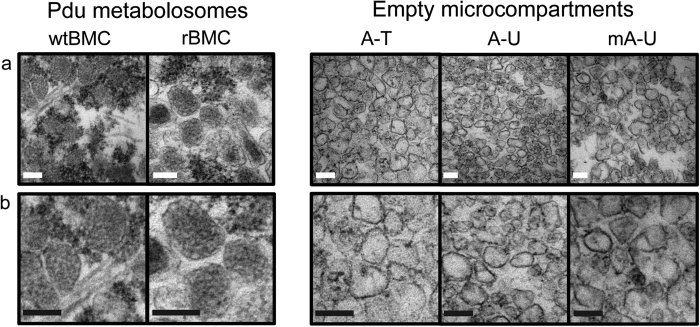
TEM of thin-sectioned BMCs. (**a**) Internal structural organization of wtBMCs, rBMCs and eBMCs (A-U, mA-U and A-T) after 70 nm sectioning. Scale bars correspond to 100 nm each. (**b**) Magnified images of samples in (**a**) showing structural organization of wtBMCs, rBMCs and eBMCs (A-U, mA-U and A-T).

To investigate and compare the internal lumen of the various BMC structures for the presence of internalized protein cargo, isolated complete BMCs (wtBMCs and rBMCs) and eBMCs (A-T, A-U, mA-U) were embedded in resin, thin sectioned and imaged by TEM (Fig. 6). When visualized, both wtBMCs and rBMCs structures showed an intact outline with a pentameric or hexameric shape. Particles appeared heterogeneous in size and shape, as described previously, and the sampled diameter and area values corresponded to the analysis shown in Fig. 4. Strikingly, the lumen of wtBMCs and rBMC appeared highly electron dense with granular spots (Fig. 6). Such density is consistent with the physiological role of Pdu microcompartments to sequester enzymes for 1,2-PD utilization. However, no higher order arrangement of internal density was observed for the wtBMC and rBMC particles as has been observed in the carboxysome^7^,^11^. In contrast, the eBMC variants (A-T, A-U, mA-U) showed intact outlines that appeared heterogeneous in size and shape as observed previously, but all had low electron density in the lumen (Fig. 6). This suggests that eBMCs form empty or hollow structures. The formation of intact and hollow structures is not affected by the presence of PduT or mCherry fused to PduA. These observations support the hypothesis that eBMCs form intact, but empty, structures.

## Discussion

In this study on the wtBMCs and their redesigned variants we have quantified structural differences between complete and empty BMCs and shown that the observed differences could be attributable to the variations in the shell protein composition of the BMC supramolecular structures. We also reveal that the presence of a protein cargo, while not required for the assembly of the BMCs, could mediate changes in the incorporation of the various shell proteins. To enable quantitative structural characterization of the BMCs, we have developed and refined purification methods in order to obtain these nano-structures in a high purity and yield. Interestingly, eBMCs purified more effectively with Y-PER™ whereas rBMCs and wild-type BMCs purified more readily with B-PER™. The differences in the purification likely reflect differences in biochemical and biophysical properties. To investigate this further, the protein composition of wtBMCs, rBMCs and the eBMCs variants (A-T, A-U and mA-U) were analyzed by a combination of 2D-PAGE and MALDI mass spectrometry. This resulted in a series of well-resolved protein spots that allowed a detailed proteomic analysis of the organelles to be undertaken. In all cases the shell proteins were identifiable, including the vertex protein PduN, whose presence had hitherto evaded identification due to its low abundance. The *C. freundii* Pdu BMC and the recombinant version produced in *E. coli* produced a similar proteomic profile to that observed with the *S. enterica* Pdu BMC^16^.

The quantification of the various shell proteins associated with BMC formation (Fig. 2 and Supplementary Table 4) provided some interesting observations. With wtBMCs and rBMCs, PduB is the most abundant shell protein. In contrast, with all eBMC variants PduA is the major shell protein. In both the complete (wtBMC and rBMC) and empty (A-T, A-U, mA-U) BMCs the major shell proteins included PduA, PduB, PduB’ and PduJ, with the remaining shell proteins (PduK, PduN, PduT, PduU) making only a minor contribution to the overall composition of the shell of the organelle. Significantly, the presence of the mCherry-PduA fusion, the most abundant BMC shell building block, did not affect the formation of the eBMC. This helped to confirm the idea that the Pdu BMC assembly is a flexible process that is able to accommodate some variation in composition but at the same time giving rise to highly stable macro-molecular assemblies.

The size, nano-mechanical properties and internal structural organization of the wtBMCs, rBMCs and eBMCs were investigated by a complementary combination of TEM and AFM. AFM has recently been reported to provide detail on a uniformly orientated shell protein sheet^42^, and has also been used to characterize a variety of virus assemblies^43^. These together with the present study suggest that AFM has the potential to provide detailed structural and mechanical information on native and complete BMCs *in situ*. Here, significant size distribution differences were observed between complete BMCs (wtBMCs and rBMCs) and empty eBMCs (A-T, A-U and mA-U) by single particle measurements. The average diameter of all tested eBMC variants was approximately half of that observed for wtBMC and rBMC particles. The average apparent cross-sectional area of eBMC variants was approximately 1/3 of the average apparent cross-sectional area of Pdu BMCs (wtBMC and rBMC). The average peak height of all eBMC variants imaged by AFM was around 40% of the average peak height of the wtBMCs and rBMCs. The nano-mechanical stability of wtBMC and rBMC structures measured by AFM-QNM were compared to the eBMC variants. This revealed that the complete BMCs (wtBMCs and rBMCs) are less stiff and more deformable than the smaller eBMCs. While these nano-mechanics data do not solely reflect the intrinsic elasticity of the shell protein material since they are also influenced by the size and shape of the BMCs, they highlight the difference in mechanical behavior of the different BMC nano-structures, and corroborates the smaller size distribution and the absence of protein cargo in eBMCs compared with the complete BMCs. Moreover, detailed imaging of thin-sectioned isolated compartments confirmed that the complete BMCs (wtBMCs and rBMCs) contain a protein cargo.

What the combined biochemical and the image data tell us is that some of the structural differences between complete and empty BMCs could be attributable to variations in the shell protein composition of the supramolecular structures. Similarly, the presence of a protein cargo could also mediate changes in the incorporation of the various shell proteins. Larger BMCs, containing a protein cargo, would also be expected to be more malleable to deformation under pressure. It may be that these two latter points are connected in that the presence of a protein cargo helps in the formation of complete, larger BMCs. For instance, the current model of BMC self-assembly, which has been deduced from a study of carboxysome biogenesis, involves a nucleation event that involves the assembly of cargo proteins prior to encapsulation of preformed shell protein faces^44^,^45^. However, in the absence of an aggregating protein cargo the shell proteins may self-assemble into smaller structures that are represented by the eBMCs.

Significantly, TEM imaging (Fig. 6) and 2D-PAGE data (Supplementary Figure 3) all suggest that the cellular cytoplasmic content appears to be excluded from the lumen of wtBMCs, rBMCs and eBMCs, during their formation^36^,^46^,^47^. Similarly, it is important to note that the absence of PduT or the inclusion of a fused fluorescent protein to PduA has no influence on eBMC formation. This supports our hypothesis that BMC formation is a stable and flexible process that can be utilized in synthetic biology for the enhanced design of nano-compartments. Indeed, engineering of chimeric BMC shells in carboxysomes has been shown to be successful^32^. Both the incorporation of protein cargo into the BMC and shell protein composition are important parameters in defining the size and shape of the BMC assemblies. Altering the shell protein composition may represent an interesting approach for tailoring of the compartment shell size to its redesigned functions. BMCs represent a model system for which novel design principles can be determined^33^,^35^. Such insights will allow the development of bioengineered internal chassis that will have application especially with metabolic pathways that involve the biosynthesis of toxic intermediates. Moreover, the ability to isolate eBMCs easily, and to unfold and refold them with a new cargo of protein or small molecule, could also allow their use as alternative delivery systems.

## Material and Methods

### Chemicals and reagents

All chemicals and reagents were purchased from Sigma unless stated otherwise.

### Bacterial Strains and Media

A complete list of all strains and plasmids used in this study is presented in Supplementary Table 5. *E. coli* BL21(DE3) and BL21(DE3)-pLysS (Promega, Southampton, UK) transformants were grown in 2YT media in presence of appropriate antibiotics (50 μg mL^−1^ ampicillin and/or 34 μg mL^−1^ chloramphenicol). All strains were grown at 37 °C to an OD_600_ of 0.4. Protein expression was induced with the addition of 100 μM IPTG and cultures were grown overnight at 19 °C prior to microcompartment purification and electron microscopy. Wild type *C. freundii* ballerup 7851 were grown in NCE media with succinate as the carbon source as described previously^41^. At an OD_600_ of 1.0, 1,2-PD (1% v/v) was added to the NCE media and the cultures were grown overnight at 19 °C before microcompartment purification. For electron microscopy 50 mL of overnight culture were resuspended in fresh NCE media containing 2% (v/v) 1,2-propanediol without succinate and grown for 1 h at 37 °C/160 rpm.

### Purification of Pdu Microcompartments

eBMCs (A-U, mA-U and A-T) were purified using yeast protein extraction reagent (Y-PER™), which included an essential salt precipitation step^35^. BMCs (rBMCs and wild type) were purified using bacterial protein extraction reagent (B-PER™). Pdu metabolosomes were separated from cell debris at 12,000× *g*. BMCs were pelleted at 20,000× *g*^41^.

### Thin sections of cells and purified microcompartments

Samples were fixed in 2 mL 2.5% (v/v) glutaraldehyde in 100 mM sodium cacodylate pH 7.2 for 2 hours, pelleted by centrifugation at 5,000× *g* for 2 min and washed twice in 100 mM sodium cacodylate pH 7.2 for 15 min. Samples were post-fixed with 1% (w/v) osmium tetroxide in 100 mM sodium cacodylate pH 7.2 for 2 h followed by two washes in dH_2_O. Samples were first dehydrated by incubation in an ethanol gradient: 50% (v/v) EtOH for 10 min, 70% (v/v) EtOH overnight, and twice in 100% (v/v) anhydrous EtOH for 10 min. This was followed by two final dehydration steps in propylene oxide for 15 min. Samples were equilibrated in 1 mL of a 1:1 propylene oxide and Agar LV Resin for 30 min. Samples were embedded in two steps of 100% Agar LV resin for 1.5 h each and resuspended in 0.5 mL Agar LV resin, centrifuged for 10 min at 3,000× *g* in Beem capsules and polymerized at 60 °C for 16 h. Samples were thin sectioned on a RMC MT-XL ultramicrotome. Sections were stained with 4.5% (w/v) uranyl acetate in 1% (v/v) acetic acid for 45 min and 0.1% (w/v) Reynolds lead citrate for 8 min^17^.

### TEM and AFM analysis of purified microcompartments

For EM analysis 10 μL of purified microcompartments were mounted onto Formvar, carbon coated 400 mesh, copper grids for 2 min, followed by the addition of 20 μL of 2.5% (v/v) glutaraldehyde in PBS for 1 min. The grids were washed three times in 20 μL drops of 2.5% (v/v) glutaraldehyde in PBS and then three times in dH_2_O. Finally, the grids were dried and stained with 2% (w/v) aqueous uranyl acetate. Grids were analyzed at zero-loss bright field mode in an energy-filtered transmission electron microscope (Jeol 1230 TEM operated at an accelerating voltage of 80kV).

For AFM, purified microcompartment samples (wtBMC, rBMC, A-U, mA-U, A-T,) were deposited onto freshly cleaved mica surfaces for five min. Microcompartments were fixed using 2.5% (v/v) glutaraldehyde in PBS for 5 min. The surface was washed three times with 1 mL sterile filtered deionized H_2_O and dried under a gentle stream of N_2_ gas. Images were collected in air using Bruker Multimode 8 scanning probe microscope (Bruker). ScanAsyst Air cantilever probes (Bruker) with a nominal spring constant of 0.4 N/m were used. Height images of 2048 × 2048 to 4096 × 4096 pixels in size, covering surface areas of 10 × 10 μm to 20 × 20 μm were acquired at 20 °C in air. Sample tilt and scanner bow were removed using NanoScope 1.40 (Bruker) as described previously^35^. Images were further analyzed using Image J 1.48 v^48^ or NanoScope 1.40. Size distribution measurements of TEM and AFM images were conducted using Image J 1.48 v. Diameters were calculated assuming a circular geometry model for the microcompartments. Maximum height information of 120 single particles for each construct were obtained using NanoScope 1.40. For AFM Peak-Force QNM experiments, a TAP525 probe was used with a peak force setpoint of 250 nN. The spring constant (116 N/m) was calculated by using Sader’s method^49^, and the deflection sensitivity (45.23 nm/V) was calculated by ramping onto a sapphire surface. The tip geometry (8.6 nm tip radius) was estimated using an absolute method with a titanium roughness surface following the standard procedure in Nanoscope 1.40. The nano-mechanical properties of twenty particles for each sample were analyzed using the DMT model, the DMT modulus and deformation was quantified (Supplementary Table 6) using NanoScope 1.40.

### 2D-PAGE

Purified microcompartments (160 μg of wtBMC, rBMC, A-U, mA-U or A-T) were used for isoelectric focusing. Strips (pH 3.0–10) were sealed on top of 15% (v/v) Tris-glycine gels, and run at 150 V for 150 min^50^. Gels were stained using Coomassie Brilliant Blue and spots were picked for MALDI ToF-ToF. The protein content was quantified relative to PduA using Image J 1.48 v of 3 independent cultures (N = 3). Standard errors of mean are given.

### In-gel digestion from MS and MS-MS analysis

Excised, diced protein gel spots were washed, reduced and S-alkylated as described previously^51^. A sufficient volume of 2 ng μL^−1^ of trypsin (modified sequencing grade, Promega, Southampton, UK) in 25 mM ammonium bicarbonate was added to cover the gel pieces and digestion was performed overnight at 20 °C. The digests were then acidified by the addition of half the volume of 50% (v/v) acetonitrile with 5% (v/v) formic acid prior to MS and MS-MS analysis^52^.

### MS and MS-MS analysis

The sample (1 μL of the above peptide digest) was placed on the sample target (AnchorChip standard, 800 μm, Bruker) and dried. 0.5 μL of matrix was added and dried. The matrix was α-cyano-4- hydroxy-cinnamic acid (α-CHCA, 0.7 mgmL^−1^ in 85% (v/v) acetonitrile, 0.1% (v/v)TFA and 1 mM NH_4_H_2_PO_4_, Sigma-Aldrich, Gillingham, UK). For external calibration in the protein mass range, Peptide Calibration Standard I (Bruker) was used. MALDI-TOF MS and MALDI TOF-TOF MS-MS analysis was performed in the positive ion mode using a Bruker UltrafleXtreme. The spectra were obtained in reflector mode with an acceleration voltage of 25 kV and a pulse ion extraction time of 80 ns. The mass range for MS was generally between 700 and 3500 m/z. The number of laser shots summed in MS was 3500. The number of laser shots summed in MS-MS was 3000. The software FlexAnalysis (Bruker, Bremen, Germany) was used for peak picking prior to using the standard Mascot search engine (Matrix Science Ltd, London, UK). The peptide mass fingerprint was searched against the NCBI proteobacteria protein database^52^.

## Additional Information

**How to cite this article**: Mayer, M. J. *et al.* Effect of bio-engineering on size, shape, composition and rigidity of bacterial microcompartments. *Sci. Rep.* **6**, 36899; doi: 10.1038/srep36899 (2016).

**Publisher’s note:** Springer Nature remains neutral with regard to jurisdictional claims in published maps and institutional affiliations.

## Supplementary Material

Supplementary Information

## Figures and Tables

**Figure 1:**
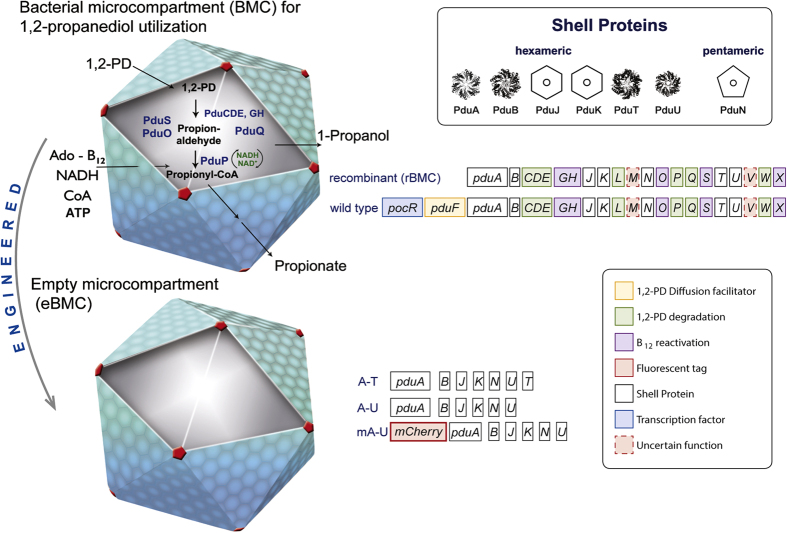
Schematic illustration of the Pdu shell and shell proteins associated with the wtBMC, rBMC and eBMC variants (A-U, mA-U and A-T). Shell Proteins: The protein structures for PduA, PduB, PduT and PduU are shown, revealing the central pores within the hexameric or pseudohexameric symmetry. Hexameric shell proteins form the facets of the structure. The pentameric vertex protein (PduN) is thought to take up the vertex position of the structure. Bacterial microcompartment (BMC) for 1,2-propanediol utilization: wtBMCs and rBMCs incorporate many of the enzymes necessary for 1,2-PD degradation as well as enzymes for adenosylcobalamin reactivation. The rBMC plasmid lacks the genes encoding for the diffusion facilitator protein (yellow, *pduF*) and the transcription factor (cyano, *pocR*). Substrate (1,2-PD) and cofactors (ATP, CoA, NAD^+^ and B_12_) are thought to diffuse through the gated pores in the shell protein. Empty microcompartment (eBMC): Three different shell protein constructs consisting of shell protein genes (A-T, A-U and mA-U) can be produced in *E. coli* (Supplementary). A-T is the basic eBMC that houses all the shell proteins (PduA, B, B’, J, K. N. U, T). A-U is composed of six Pdu shell protein, PduA, B, B’, J, K, N, U. The mA-U construct has a C-terminal fusion of mCherry (red, fluorescent tag) to PduA and thus consists of mCherry-PduA, B, B’, J, K, N, U.

**Table 1:** Image analyses of microcompartment size distributions.

		Diameter/nm	Area/μm^2^	Height/nm
wtBMC	TEM	127 ± 13.2	0.0128 ± 0.0026	
	AFM	131 ± 15.4	0.0137 ± 0.0032	49.99 ± 8.10
rBMC	TEM	122 ± 26.4	0.0122 ± 0.0053	
	AFM	133 ± 23.6	0.0143 ± 0.0053	50.15 ± 8.36
A-T	TEM	77 ± 18.5	0.0049 ± 0.0024	
	AFM	59 ± 7.9	0.0028 ± 0007	18.74 ± 3.31
A-U	TEM	65 ± 7.4	0.0034 ± 0.0008	
	AFM	66 ± 8.4	0.0034 ± 0.0009	18.42 ± 2.41
mA-U	TEM	74 ± 23	0.0047 ± 0.0033	
	AFM	56 ± 8.0	0.0025 ± 0.0007	18.46 ± 3.16

Values denote mean ± SD.

## References

[b1] Bobik T. A., Lehman B. P. & Yeates T. O. Bacterial microcompartments: widespread prokaryotic organelles for isolation and optimization of metabolic pathways. Mol Microbiol 98, 193–207 (2015). 26148529 10.1111/mmi.13117 PMC4718714

[b2] Kerfeld C. A., Heinhorst S. & Cannon G. C. Bacterial microcompartments. Annu Rev Microbiol 64, 391–408 (2010). 20825353 10.1146/annurev.micro.112408.134211

[b3] Yeates T. O., Kerfeld C. A., Heinhorst S., Cannon G. C. & Shively J. M. Protein-based organelles in bacteria: carboxysomes and related microcompartments. Nature Reviews Microbiology 6, 681–691 (2008). 18679172 10.1038/nrmicro1913

[b4] Bobik T. A., Havemann G. D., Busch R. J., Williams D. S. & Aldrich H. C. The propanediol utilization (pdu) operon of Salmonella enterica serovar Typhimurium LT2 includes genes necessary for formation of polyhedral organelles involved in coenzyme B(12)-dependent 1, 2-propanediol degradation. Journal of Bacteriology 181, 5967–5975 (1999). 10498708 10.1128/jb.181.19.5967-5975.1999 PMC103623

[b5] Shively J. M., Ball F., Brown D. H. & Saunders R. E. Functional organelles in prokaryotes: polyhedral inclusions (carboxysomes) of Thiobacillus neapolitanus. Science 182, 584–586 (1973). 4355679 10.1126/science.182.4112.584

[b6] Shively J. M., Bock E., Westphal K. & Cannon G. C. Icosahedral inclusions (carboxysomes) of Nitrobacter agilis. Journal of Bacteriology 132, 673–675 (1977). 199579 10.1128/jb.132.2.673-675.1977 PMC221910

[b7] Iancu C. V. *et al.* The Structure of Isolated Synechococcus Strain WH8102 Carboxysomes as Revealed by Electron Cryotomography. Journal of Molecular Biology 372, 764–773 (2007). 17669419 10.1016/j.jmb.2007.06.059 PMC2453779

[b8] Iancu C. V. *et al.* Organization, structure, and assembly of α-carboxysomes determined by electron cryotomography of intact cells. Journal of Molecular Biology 396, 105–117 (2010). 19925807 10.1016/j.jmb.2009.11.019 PMC2853366

[b9] Kerfeld C. A. *et al.* Protein structures forming the shell of primitive bacterial organelles. Science 309, 936–938 (2005). 16081736 10.1126/science.1113397

[b10] Tanaka S. *et al.* Atomic-level models of the bacterial carboxysome shell. Science 319, 1083–1086 (2008). 18292340 10.1126/science.1151458

[b11] Schmid M. F. *et al.* Structure of Halothiobacillus neapolitanus carboxysomes by cryo-electron tomography. Journal of Molecular Biology 364, 526–535 (2006). 17028023 10.1016/j.jmb.2006.09.024 PMC1839851

[b12] Brinsmade S. R., Paldon T. & Escalante-Semerena J. C. Minimal functions and physiological conditions required for growth of salmonella enterica on ethanolamine in the absence of the metabolosome. J Bacteriol 187, 8039–8046 (2005). 16291677 10.1128/JB.187.23.8039-8046.2005 PMC1291257

[b13] Badger M. R. & Price G. D. CO2 concentrating mechanisms in cyanobacteria: molecular components, their diversity and evolution. J Exp Bot 54, 609–622 (2003). 12554704 10.1093/jxb/erg076

[b14] Axen S. D., Erbilgin O. & Kerfeld C. A. A Taxonomy of bacterial microcompartment loci constructed by a novel scoring method. PLoS Computational Biology 10, e1003898 (2014). 25340524 10.1371/journal.pcbi.1003898 PMC4207490

[b15] Cheng S., Sinha S., Fan C., Liu Y. & Bobik T. A. Genetic analysis of the protein shell of the microcompartments involved in coenzyme B12-dependent 1,2-propanediol degradation by Salmonella. Journal of Bacteriology 193, 1385–1392 (2011). 21239588 10.1128/JB.01473-10 PMC3067621

[b16] Havemann G. D. & Bobik T. A. Protein content of polyhedral organelles involved in coenzyme B12-dependent degradation of 1,2-propanediol in Salmonella enterica serovar Typhimurium LT2. Journal of Bacteriology 185, 5086–5095 (2003). 12923081 10.1128/JB.185.17.5086-5095.2003 PMC180998

[b17] Parsons J. B. *et al.* Biochemical and structural insights into bacterial organelle form and biogenesis. Journal of Biological Chemistry 283, 14366–14375 (2008). 18332146 10.1074/jbc.M709214200

[b18] Johnson C. L. *et al.* Functional genomic, biochemical, and genetic characterization of the Salmonella pduO gene, an ATP:cob(I)alamin adenosyltransferase gene. Journal of Bacteriology 183, 1577–1584 (2001). 11160088 10.1128/JB.183.5.1577-1584.2001 PMC95042

[b19] Parsons J. B. *et al.* Characterisation of PduS, the pdu metabolosome corrin reductase, and evidence of substructural organisation within the bacterial microcompartment. PLoS One 5, e14009 (2010). 21103360 10.1371/journal.pone.0014009 PMC2982820

[b20] Sampson E. M., Johnson C. L. & Bobik T. A. Biochemical evidence that the pduS gene encodes a bifunctional cobalamin reductase. Microbiology 151, 1169–1177 (2005). 15817784 10.1099/mic.0.27755-0

[b21] Sampson E. M. & Bobik T. A. Microcompartments for B12-dependent 1,2-propanediol degradation provide protection from DNA and cellular damage by a reactive metabolic intermediate. Journal of Bacteriology 190, 2966–2971 (2008). 18296526 10.1128/JB.01925-07 PMC2293232

[b22] Cai F. *et al.* The structure of CcmP, a tandem bacterial microcompartment domain protein from the beta-carboxysome, forms a subcompartment within a microcompartment. Journal of Biological Chemistry 288, 16055–16063 (2013). 23572529 10.1074/jbc.M113.456897 PMC3668761

[b23] Crowley C. S. *et al.* Structural insight into the mechanisms of transport across the Salmonella enterica Pdu microcompartment shell. J Biol Chem 285, 37838–37846 (2010). 20870711 10.1074/jbc.M110.160580 PMC2988387

[b24] Crowley C. S., Sawaya M. R., Bobik T. A. & Yeates T. O. Structure of the PduU Shell Protein from the Pdu Microcompartment of Salmonella. Structure 16, 1324–1332 (2008). 18786396 10.1016/j.str.2008.05.013 PMC5878062

[b25] Heldt D. *et al.* Structure of a trimeric bacterial microcompartment shell protein, EtuB, associated with ethanol utilization in Clostridium kluyveri. Biochem J 423, 199–207 (2009). 19635047 10.1042/BJ20090780

[b26] Klein M. G. *et al.* Identification and structural analysis of a novel carboxysome shell protein with implications for metabolite transport. Journal of Molecular Biology 392, 319–333 (2009). 19328811 10.1016/j.jmb.2009.03.056

[b27] Pang A., Liang M., Prentice M. B. & Pickersgill R. W. Substrate channels revealed in the trimeric Lactobacillus reuteri bacterial microcompartment shell protein PduB. Acta Crystallographica Section D: Biological Crystallography 68, 1642–1652 (2012). 23151629 10.1107/S0907444912039315

[b28] Pang A., Warren M. J. & Pickersgill R. W. Structure of PduT, a trimeric bacterial microcompartment protein with a 4Fe-4S cluster-binding site. Acta Crystallographica Section D: Biological Crystallography 67, 91–96 (2011). 21245529 10.1107/S0907444910050201

[b29] Tanaka S., Sawaya M. R. & Yeates T. O. Structure and mechanisms of a protein-based organelle in Escherichia coli. Science 327, 81–84 (2010). 20044574 10.1126/science.1179513

[b30] Wheatley N. M., Gidaniyan S. D., Liu Y., Cascio D. & Yeates T. O. Bacterial microcompartment shells of diverse functional types possess pentameric vertex proteins. Protein Science 22, 660–665 (2013). 23456886 10.1002/pro.2246 PMC3649267

[b31] Cai F. *et al.* The pentameric vertex proteins are necessary for the icosahedral carboxysome shell to function as a CO2 leakage barrier. PLoS One 4, e7521 (2009). 19844578 10.1371/journal.pone.0007521 PMC2760150

[b32] Cai F., Sutter M., Bernstein S. L., Kinney J. N. & Kerfeld C. A. Engineering bacterial microcompartment shells: Chimeric shell proteins and chimeric carboxysome shells. ACS Synthetic Biology 4, 444–453 (2014). 25117559 10.1021/sb500226j

[b33] Frank S., Lawrence A. D., Prentice M. B. & Warren M. J. Bacterial microcompartments moving into a synthetic biological world. Journal of Biotechnology 163, 273–279 (2013). 22982517 10.1016/j.jbiotec.2012.09.002

[b34] Chowdhury C. *et al.* Selective molecular transport through the protein shell of a bacterial microcompartment organelle. Proc Natl Acad Sci USA 112, 2990–2995 (2015). 25713376 10.1073/pnas.1423672112 PMC4364225

[b35] Lawrence A. D. *et al.* Solution structure of a bacterial microcompartment targeting peptide and its application in the construction of an ethanol bioreactor. ACS Synthetic Biology 3, 454–465 (2014). 24933391 10.1021/sb4001118 PMC4880047

[b36] Parsons J. B. *et al.* Synthesis of empty bacterial microcompartments, directed organelle protein incorporation, and evidence of filament-associated organelle movement. Molecular Cell 38, 305–315 (2010). 20417607 10.1016/j.molcel.2010.04.008

[b37] Held M. *et al.* Engineering formation of multiple recombinant Eut protein nanocompartments in E. coli. Sci Rep 6, 24359 (2016). 27063436 10.1038/srep24359 PMC4827028

[b38] Quin M. B., Perdue S. A., Hsu S. Y. & Schmidt-Dannert C. Encapsulation of multiple cargo proteins within recombinant Eut nanocompartments. Appl Microbiol Biotechnol (2016). 10.1007/s00253-016-7737-8 27450681

[b39] Bonacci W. *et al.* Modularity of a carbon-fixing protein organelle. Proceedings of the National Academy of Sciences of the United States of America 109, 478–483 (2012). 22184212 10.1073/pnas.1108557109 PMC3258634

[b40] Sriramulu D. D. *et al.* Lactobacillus reuteri DSM 20016 produces cobalamin-dependent diol dehydratase in metabolosomes and metabolizes 1,2-propanediol by disproportionation. J Bacteriol 190, 4559–4567 (2008). 18469107 10.1128/JB.01535-07 PMC2446795

[b41] Sinha S., Cheng S., Fan C. & Bobik T. A. The PduM protein is a structural component of the microcompartments involved in coenzyme B(12)-dependent 1,2-propanediol degradation by Salmonella enterica. Journal of Bacteriology 194, 1912–1918 (2012). 22343294 10.1128/JB.06529-11 PMC3318458

[b42] Sutter M. *et al.* Visualization of Bacterial Microcompartment Facet Assembly Using High-Speed Atomic Force Microscopy. Nano letters (2015). 10.1021/acs.nanolett.5b04259 PMC4789755 26617073

[b43] Mateu M. G. Mechanical properties of viruses analyzed by atomic force microscopy: a virological perspective. Virus Res 168, 1–22 (2012). 22705418 10.1016/j.virusres.2012.06.008

[b44] Cameron J. C., Wilson S. C., Bernstein S. L. & Kerfeld C. A. Biogenesis of a bacterial organelle: the carboxysome assembly pathway. Cell 155, 1131–1140 (2013). 24267892 10.1016/j.cell.2013.10.044

[b45] Chen A. H., Robinson-Mosher A., Savage D. F., Silver P. A. & Polka J. K. The bacterial carbon-fixing organelle is formed by shell envelopment of preassembled cargo. PloS One 8, e76127 (2013). 24023971 10.1371/journal.pone.0076127 PMC3762834

[b46] Lassila J. K., Bernstein S. L., Kinney J. N., Axen S. D. & Kerfeld C. A. Assembly of robust bacterial microcompartment shells using building blocks from an organelle of unknown function. Journal of Molecular Biology 426, 2217–2228 (2014). 24631000 10.1016/j.jmb.2014.02.025

[b47] Sargent F. *et al.* A synthetic system for expression of components of a bacterial microcompartment. Microbiology 159, 2427–2436 (2013). 24014666 10.1099/mic.0.069922-0 PMC3836489

[b48] Schneider C. A., Rasband W. S. & Eliceiri K. W. NIH Image to ImageJ: 25 years of image analysis. Nature methods 9, 671–675 (2012). 22930834 10.1038/nmeth.2089 PMC5554542

[b49] Sader J. E., Pacifico J., Green C. P. & Mulvaney P. General scaling law for stiffness measurement of small bodies with applications to the atomic force microscope. Journal of Applied Physics 97, 124903 (2005).

[b50] Hogwood C. E. M., Tait A. S., Koloteva‐Levine N., Bracewell D. G. & Smales C. M. The dynamics of the CHO host cell protein profile during clarification and protein A capture in a platform antibody purification process. Biotechnology and Bioengineering 110, 240–251 (2013). 22806637 10.1002/bit.24607

[b51] Fox K., Fox A., Rose J. & Walla M. Speciation of coagulase negative staphylococci, isolated from indoor air, using SDS PAGE gel bands of expressed proteins followed by MALDI TOF MS and MALDI TOF-TOF MS-MS analysis of tryptic peptides. Journal of Microbiological Methods 84, 243–250 (2011). 21167877 10.1016/j.mimet.2010.12.007

[b52] Alanen H. I. *et al.* Efficient export of human growth hormone, interferon α2b and antibody fragments to the periplasm by the Escherichia coli Tat pathway in the absence of prior disulfide bond formation. Biochimica et Biophysica Acta (BBA)-Molecular Cell Research 3, 756–763 (2014). 10.1016/j.bbamcr.2014.12.027 25554517

