# Supplementary material for: Effect of bio-engineering on size, shape, composition and rigidity of bacterial microcompartments
Source: Sci Rep. 2016 Nov 15;6:36899. doi: 10.1038/srep36899 (PMC5109269; doi:10.1038/srep36899)
Supplement: Supplementary Information [file srep36899-s1.pdf]

Supplementary Information for:

Effect of bio-engineering on size, shape, composition and rigidity of bacterial microcompartments.

Matthias J. Mayer[1], Rokas Juodeikis[1], Ian R. Brown[1], Stefanie Frank[2], David J. Palmer[1], Evelyne Deery[1], David M. Beal[1], Wei-Feng Xue[1], Martin J. Warren[1]**

1. Centre for Molecular Processing and School of Biosciences, University of Kent, Giles Lane, Canterbury, Kent CT2 7NJ, UK

2. Department of Biochemical Engineering, University College London, Bernard Katz Building, Gordon Street, London WC1E 6BT, UK

*Corresponding Authors: Martin J. Warren email: M.J.Warren@kent.ac.uk, or Wei-Feng Xue email: W.F.Xue@kent.ac.uk

TEM of cells forming Pdu metabolosomes and empty microcompartments

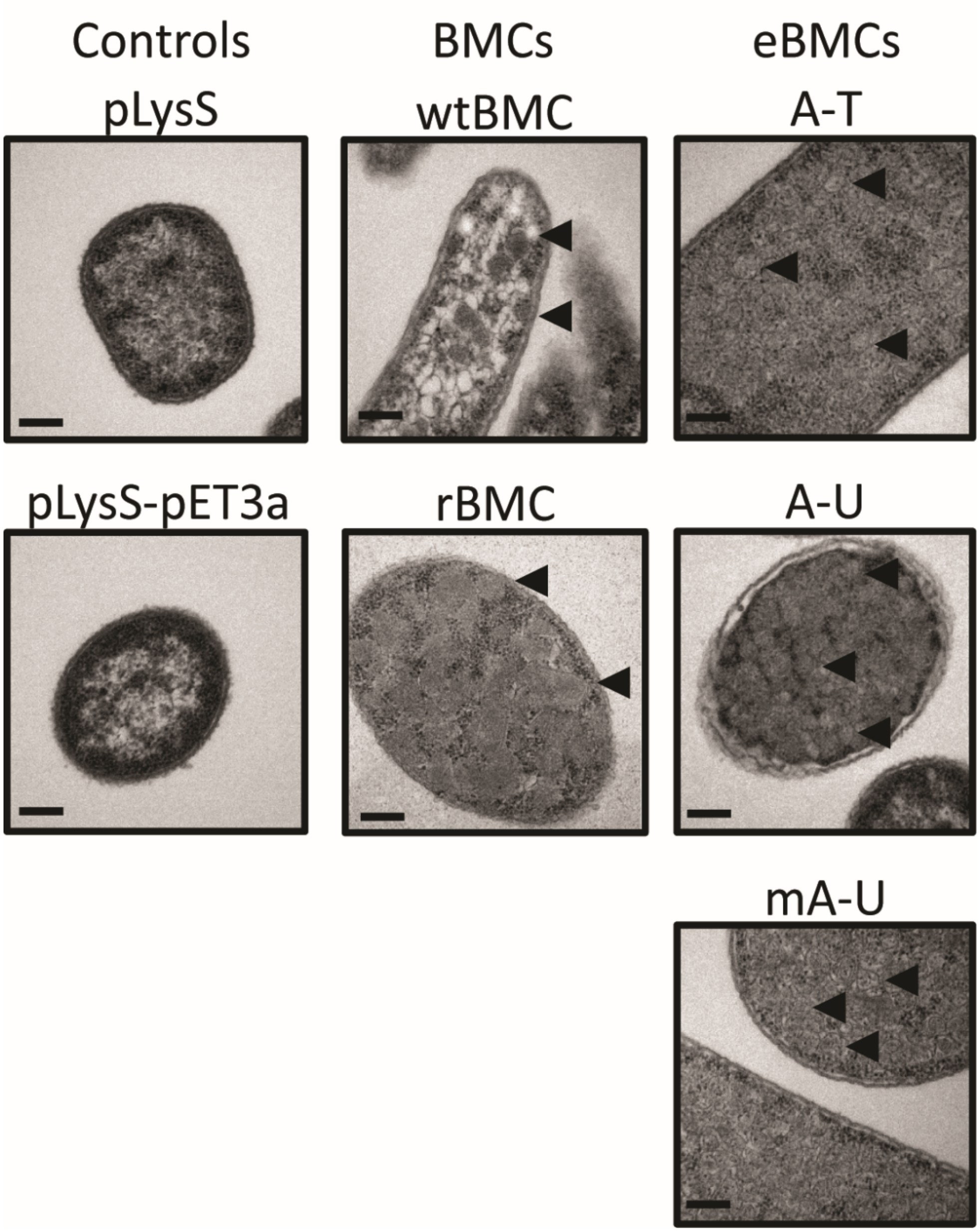

Figure 1. Electron microscopy images of strains transformed with control vectors (pLysS, pLysS-pET3a), wild-type *C. freundii,* recombinant operon (rBMC) and empty microcompartment constructs (eBMCs: A-T, A-U and A-T) [17, 19, 35, 38]. Scale bars correspond to 200 nm.

Purification of empty Pdu microcompartments (eBMC) and Pdu metabolosomes (BMC)

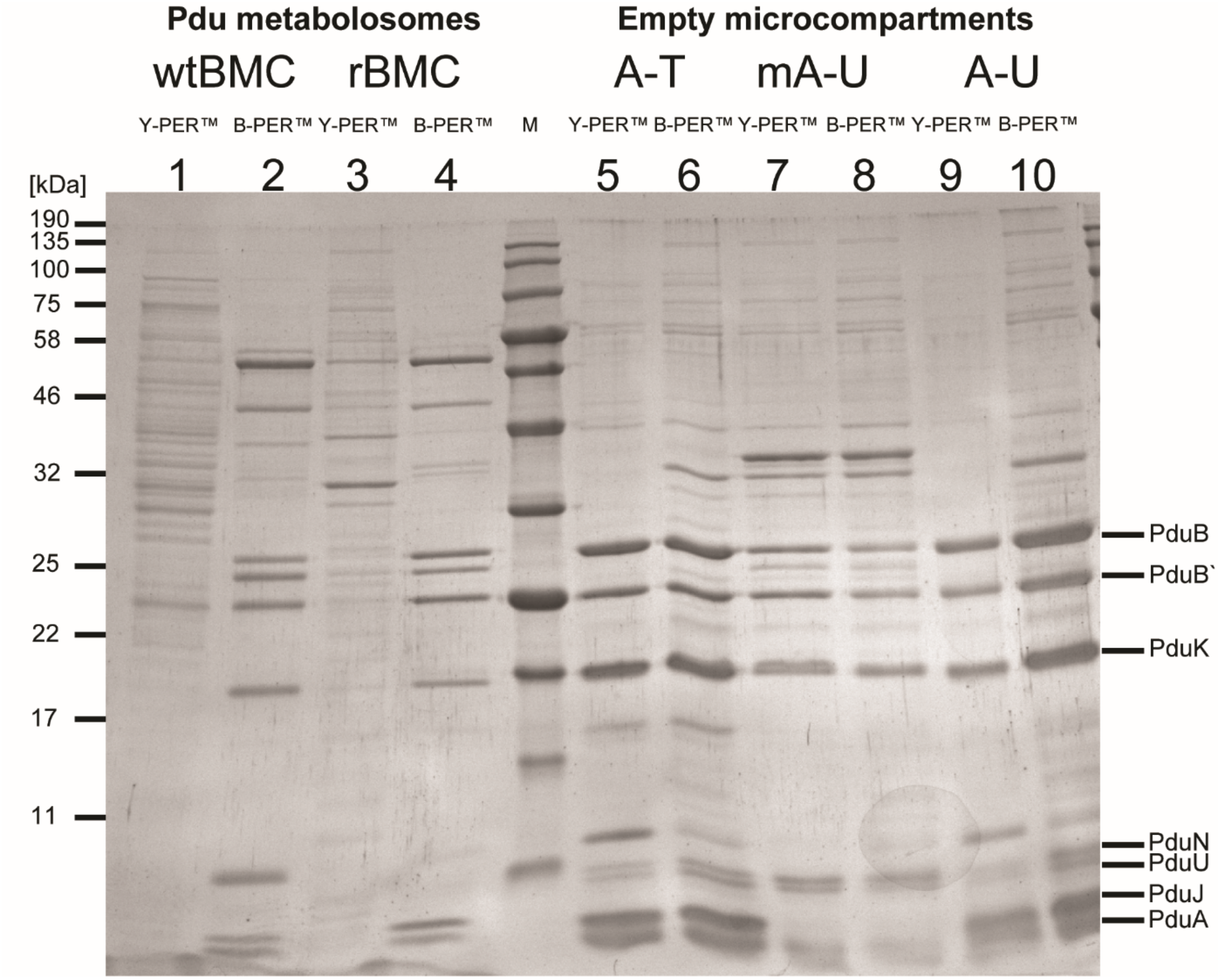

Figure 2. Summary of microcompartment purification. A 15 % polyacrylamide gel is show. 10 μl samples were loaded in each lane, (M) represents the marker lane. The gels show that empty microcompartment constructs (A-T, mA-U and A-U) can be purified (lanes 5-10) using previously published methods using bacterial protein extraction reagent (B-PER™) or yeast extraction reagent (Y-PER™). Metabolosomes (rBMC and wild type BMC) did not purify using the Y-PER™ method (lanes 1 and 3) but purified with B-PER™ (lanes 2 and 4). The expected molecular mass of the shell proteins are as follows: PduB = 28 KDa, PduB' = 23 KDa, PduK = 16 KDa, PduN = 9.2 KDa, PduU = 12.5 KDa, PduJ = 9.0 KDa, PduA = 9.5 KDa.

MALDI MS-MS

The following figure shows 2D-PAGE gels with the corresponding protein spots identified by tandem mass spectrometry consistent with previously reported proteomic data [16, 38].

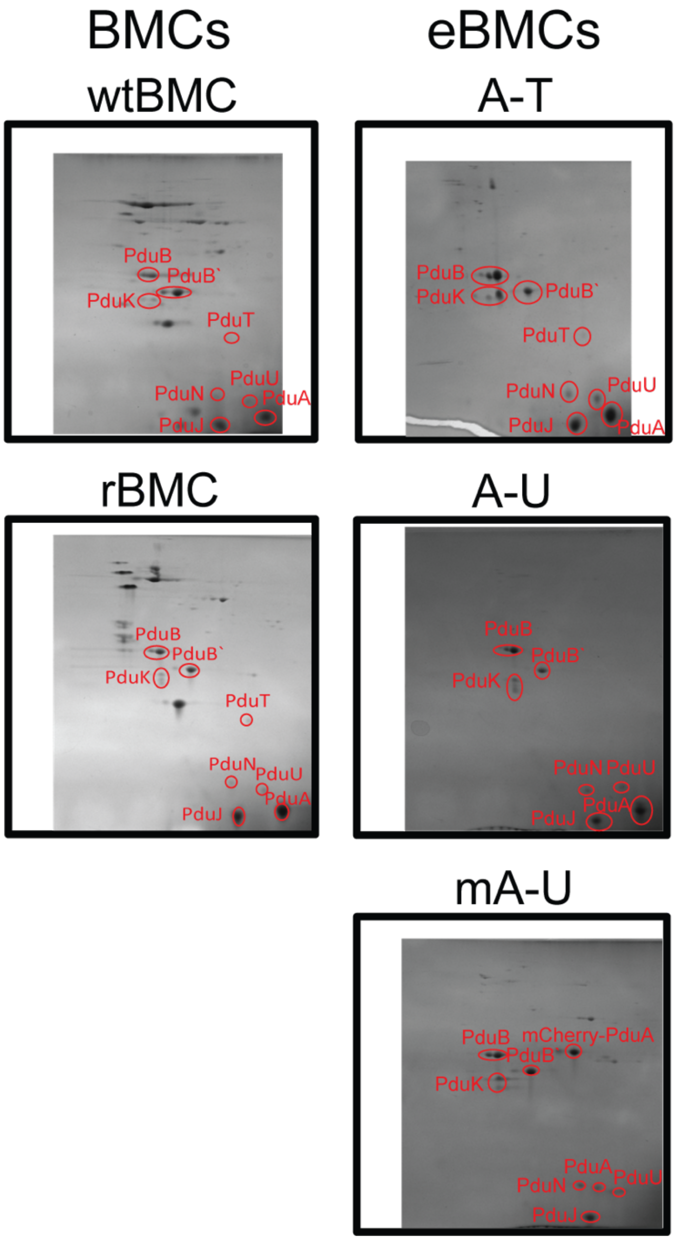

Figure 3. 2D-PAGE gels of purified Pdu microcompartments. Shell protein spots identified by MALDI TOF and TOF-TOF are shown in red.

Single particle analysis (diameter/area/height):

Table 1. The following table comprise the statistical analysis for diameter [nm] studies. Average, minimum, maximum, median and standard deviation for Pdu metabolosomes (wtBMC, rBMC) and empty microcompartments (A-T, A-U and mA-U) and. TEM and AFM values are given each.

	**Pdu metabolosomes**				**Empty microcompartments**					
**Diameter [nm]**	**wtBMC**		**rBMC**		**A-T**		**A-U**		**mA-U**	
	**TEM**	**AFM**	**TEM**	**AFM**	**TEM**	**AFM**	**TEM**	**AFM**	**TEM**	**AFM**
**Average**	127	131	122	133	77	59	65	66	74	56
**Minimum**	94	94	72	80	17	36	50	49	38	36
**Maximum**	162	175	201	202	126	80	84	96	186	80
**Median**	127	129	117	129	75	62	65	64	68	50
**Standard deviation**	13.2	15.4	26.4	23.6	18.5	7.9	7.4	8.4	23.0	8.0

Table 2. The following table comprise the statistical analysis for area [μm^2] studies. Mean, minimum, maximum, median and standard deviation are given for Pdu metabolosomes (wtBMC and rBMC) and empty microcompartments (A-T, A-U and mA-U). TEM and AFM values are given each.

Area [μm^2]	Pdu metabolosomes				Empty microcompartments					
	wtBMC		rBMC		A-T		A-U		mA-U	
	EM	AFM	EM	AFM	EM	AFM	EM	AFM	EM	AFM
**Average**	0.0128	0.0137	0.0122	0.0143	0.0049	0.0028	0.0034	0.0034	0.0047	0.0025
**Minimum**	0.0070	0.0070	0.0041	0.0050	0.0002	0.0010	0.0019	0.0019	0.0011	0.0010
**Maximum**	0.0205	0.0240	0.0316	0.0320	0.0124	0.0050	0.0056	0.0073	0.0272	0.0050
**Median**	0.0126	0.0130	0.0108	0.0130	0.0044	0.0030	0.0033	0.0032	0.0036	0.0020
**Standard Deviation**	0.0026	0.0032	0.0053	0.0053	0.0024	0.0007	0.0008	0.0009	0.0033	0.0007

Table 3. The following table comprise the statistical analysis particle height [nm] studies. Average, minimum, maximum, median and standard deviation are given for Pdu metabolosomes (wtBMC and rBMC) and empty microcompartments (A-T, A-U and mA-U). AFM values are given each.

	**Pdu metabolosomes**		**Empty microcompartments**		
	**wtBMC**	**rBMC**	**A-T**	**A-U**	**mA-U**
**Height [nm]**	**AFM**	**AFM**	**AFM**	**AFM**	**AFM**
**Average**	49.99	50.15	18.74	18.42	18.46
**Minimum**	36.00	36.00	12.00	13.00	13.50
**Maximum**	78.00	71.00	30.00	24.50	31.50
**Median**	50.00	49.00	18.00	19.00	18.30
**Standard Deviation**	8.10	8.36	3.31	2.41	3.16

Shell protein composition in empty microcompartments and Pdu metabolosomes

Table 4. Shell protein composition of studied Pdu microcompartment types (wtBMC, rBMC, A-T, A-U, mA-U). Mean values of three independent repeats (N=3) and standard error of mean are given.

		Major shell protein components				Minor shell protein components			
		**PduA**	**PduB**	**PduB`**	**PduJ**	**PduK**	**PduN**	**PduT**	**PduU**
**Pdu metabolosomes**	**wtBMC**	19.4 ± 0.9	18.4 ± 0.7	30.7 ± 1.5	23.8 ± 1.1	4.2 ± 0.3	2 ± 0.4		1.4 ± 0.2
	**rBMC**	22.5 ± 0.2	24.9 ± 0.7	27.7 ± 1.7	18.9 ± 0.1	2.4 ± 0.6	2 ± 0.2		1.4 ± 0.4
**Empty microcompartments**	**A-T**	35.9 ± 3.6	18.3 ± 0.7	12.2 ± 2.7	18.1 ± 1.0	6.6 ± 1.4	3.4 ± 0.3	1.9 ± 0.1	3.6 ± 0.9
	**A-U**	27.1 ± 4.0	23.6 ± 1.2	16.0 ± 1.4	18.2 ± 1.5	6.0 ± 1.3	5.4 ± 1.4		3.7 ± 1.1
	**mA-U**	35.3 ± 2.8	18.4 ± 1.7	13.5 ± 1.5	13 ± 1.7	7 ± 0.8	5.2 ± 0.9		7.5 ± 2.4

Strain list

Table 5. Summary of the used strains, bacterial strain, plasmid construct and source/references are noted.

	**Bacterial strain**	**Plasmid**	**Source/Reference**
Controls	Bl21(DE3)	pLysS	Novagen
	Bl21(DE3)	pLysS, pET3a	Novagen
Pdu metabolosomes	*Citrobacter freundii* ballerup 7851	wtBMC	Provided by the Sanger Institute (Cambridge, United Kingdom)
	Bl21(DE3)-plysS	pET14b-Pdu65 (A-X), rBMC	Parsons *et al.*, 2008
Empty microcompartments	Bl21(DE3)	pLysSA-U(*pduA-B-B`-J-K-N-U*), A-U	Parsons *et al.*, 2010
	Bl21(DE3)	pLysSmCherryA-U(*mcherrypduA-B-J-K-N-U*), mA-U	Parsons *et al.*, 2010
	Bl21(DE3)-plysS	pET3aA-T (*pduA-B-J-K-N-U-T*), A-T	Parsons *et al.*, 2010

Quantitative nano-mechanical mapping

Table 6. Quantitative nano-mechanical mapping at 250 nN did change the observed particle height of individual Pdu microcompartments (wtBMC, rBMC, A-T, A-U and mA-U). Mean values and standard error of mean (of 20 measured particles) are reported of, reduced DMT modulus (E*) [GPa] and deformation.

	**Construct**	**Reduced DMT modulus (E*) [GPa]**	**Deformation [nm]**
**Pdu metabolosomes**	wild type	5.4 ± 0.9	2.9 ± 0.1
	rBMC	5.0 ± 0.9	3.0 ± 0.1
**Empty microcompartments**	A-T	25.3 ± 2.2	2.01 ± 0.1
	A-U	22.8 ± 2.3	1.85 ± 0.1
	mA-U	29.8 ± 3.8	1.68 ± 0.1
